# BARRIERS AND FACILITATORS OF PARTICIPATORY ACTION RESEARCH WITH LGBTQ+ ELDERS OF COLOR DURING COVID-19

**DOI:** 10.1093/geroni/igac059.461

**Published:** 2022-12-20

**Authors:** Austin Oswald

**Affiliations:** Goldsen Institute of the University of Washington , Seattle, Washington, United States

## Abstract

Participatory action research (PAR) is a promising approach to engage older adults in self-determined research that influences policies and practices; however, the voices of older adults are very often missing from most PAR projects. The purpose of this paper is to reflect upon the challenges and opportunities of engaging lesbian, gay, bisexual, transgender, and queer (LGBTQ+) elders of color in PAR during the Covid-19 pandemic. Data were generated through interviews with LGBTQ+ elders of color and their caregivers and analyzed thematically. Findings illustrate several barriers and facilitators of conducting PAR during the pandemic. Barriers included feelings of distrust toward research(ers), pandemic related issues and illnesses, and competing priorities and interests. Facilitators included leveraging community connections and participating in community-based activities. Study findings have the potential to advance a critical turn in gerontology that is committed to the liberation of marginalized older adults in aging research, policies, and practices.

